# Efficacy analysis of combined detection of 5 Serological Tumor markers including MIF and PIVKA-II for early diagnosis of Primary Hepatic Cancer

**DOI:** 10.12669/pjms.37.5.4264

**Published:** 2021

**Authors:** Li Huan

**Affiliations:** Li Huan Department of Clinical Laboratory, Tangshan People’s Hospital, Tangshan, Hebei, 063000, China

**Keywords:** Abnormal prothrombin, AFP, AFP-13, MIF, Primary liver cancer, GP 73

## Abstract

**Objectives:**

To investigate the efficacy of combined detection of 5 serological tumor markers including macrophage migration inhibitory factor (MIF) and abnormal prothrombin (PIVKA-II) in the early diagnosis of primary liver cancer.

**Methods:**

A total of 90 patients with suspected primary liver cancer admitted to our hospital from January 2016 to May 2017 were selected as the research subjects. All patients were examined by imaging and histopathology. Enzyme-linked immunosorbent assay (ELISA) was used to detect serum MIF, GP73 and PIVKA-II. Automatic electrochemiluminescence immunoassay system was used to detect serum AFP and AFP-L3. The diagnostic value of single and combined detection of five serological tumor markers for primary liver cancer was compared and analyzed.

**Results:**

Of the 90 suspected patients with primary liver cancer, thirty-seven were excluded and 53 were confirmed. From serum MIF diagnosis, fifty-three patients had positive results for primary liver cancer, of which eight had false positive results, with a sensitivity of 84.91%, a specificity of 78.38%, and an accuracy of 82.22%, respectively. From serum GP73 diagnosis, fifty-six patients had positive results for primary liver cancer, of which 10 had false positive results, with a sensitivity of 86.79%, a specificity of 72.97%, and an accuracy of 81.11%, respectively. From serum PIVKA-II diagnosis, 48 patients had positive results for primary liver cancer, of which seven had false positive results, with the sensitivity of 77.36%, the specificity of 81.08%, and the accuracy of 78.89%, respectively. From serum AFP-L3 diagnosis, fifty-two patients had positive results for primary liver cancer, of which nine had false positive results, with a sensitivity of 81.13%, a specificity of 75.68%, and an accuracy of 78.89% respectively. From serum AFP diagnosis, 57 patients had positive results for primary liver cancer, of which seven had false positive results, with a sensitivity of 83.02%, the specificity of 81.08%, and an accuracy of 82.22%, respectively. From the combined diagnosis of 5 serological tumor markers, fifty-three patients had positive results for primary liver cancer, of which one had a false positive result, with a sensitivity of 98.11%, a specificity of 97.30%, and an accuracy of 97.78%, respectively. Combined diagnosis has significantly higher sensitivity, specificity and accuracy than a single diagnosis (P<0.05).

**Conclusion:**

Serum MIF, GP73, PIVKA-II, AFP-L3 and AFP all have certain diagnostic value for primary liver cancer; the combined detection of five serological tumor markers can significantly improve the sensitivity, specificity and accuracy of the diagnosis of primary liver cancer, with higher diagnostic value.

## INTRODUCTION

Primary liver cancer is characterized by “the most difficult to find, the most difficult to diagnose, the most difficult to treat, the fastest progress, and having the worst prognosis”. Currently, diagnosing primary liver cancer in clinic practices mainly depends on imaging and pathological examinations. The vast majority of patients with liver cancer are in the middle and advanced stages when diagnosed, lack effective treatment, and have a poor prognosis.^1^,^2^ Therefore, it is particularly important to find and investigate diagnostic indicators with enhanced sensitivity or specificity.

Serum macrophage migration inhibitory factor (MIF), Golgi protein 73 (GP73), abnormal prothrombin (protein induced by vitamin K absence or antagonist-II, PIVKA-II), alpha-fetoprotein (AFP) and lentil lectin-reactive alpha-fetoprotein-L3 (AFP-L3), as detection indicators in clinical practices, have certain diagnostic value for the early diagnosis of primary liver cancer.^2^-^4^ There have been studies of using a single one for detection, as well as combining 2 or three for detection, but whether combining 5 to improve sensitivity, specificity, and accuracy remains to be studied. In this study, the levels of MIF, GP73, PIVKA-II, AFP-L3 and AFP in serum were examined, aiming to investigate its application value in diagnosing primary liver cancer and provide a new direction for the clinical diagnosis and treatment of primary liver cancer.

## METHODS

A total of 90 patients with suspected primary liver cancer admitted to our hospital from January 2016 to May 2017 were selected as the subjects, and all underwent imaging and histopathological examinations.

### Ethical approval

The study was approved by the Institutional Ethics Committee of Tangshan People’s Hospital [No: RMYY-LLKS-2020-070 (Date: December 15,2020)], and is in accordance with the Declaration of Helsinki.

### Inclusion criteria:

1. Conformed to the diagnostic criteria concerned for primary liver cancer.^5^
2. Be diagnosed by imaging, surgical pathology, etc.
3. Had complete clinical data.
4. Be informed and signed the consent form.

### Exclusion criteria:

1. Had other malignant tumors.
2. Had underlying diseases of other important organs.
3. Had received surgical resection of tumors and treatments such as biological targeted drugs.

Five mL of venous blood was collected from all subjects, centrifuged at 2500 r/min for 10 min, the upper serum was taken and stored in a refrigerator at -80^0^C for later use. The enzyme-linked immunosorbent assay (ELISA) was used to detect serum MIF, GP73, and PIVKA-II levels, in strict accordance with the instructions of the MIF ELISA kit (product No.: ML060008, Shanghai mlbio Co., Ltd.), GP73 ELISA kit (product No.: ML038317, Shanghai mlbio Co., Ltd.), and PIVKA-II ELISA kit (product No.: JL19709-48T, Shanghai Jianglaibio Co., Ltd.) for the experimental operation, with MIF≥9.23 ng/mL, GP73≥150 μg/L and PIVKA-II > 40 mAu /mL considered positive.

An automatic electrochemiluminescence immunoassay system (model: E170, Roche, Switzerland) was used to detect serum AFP, with AFP ≥ 20 μg/L being considered positive. A micro-spin-column was used to separate AFP-L3, the AFP-L3 was detected, with AFP-L3≥10% being considered positive.From the combined detection, the result shall be considered positive in case that one has a positive result.

### Statistical Analysis

Statistical software SPSS 20.0 was used to process the data. Counting data was represented by cases (n), using the chi-square test. Measurement data were expressed as mean ± standard deviation, and an independent sample *t* test was performed for the homogeneity of variance between the 2 groups. P<0.05 was considered statistically significant.

## RESULTS

Ninety patients with suspected primary liver cancer were examined by imaging and pathology, finally 37 with primary liver cancer were finally excluded and 53 with primary liver cancer were confirmed. There was no statistically significant difference in age, gender, and body mass index (BMI) (P>0.05). Table-I.

**Table-I:** Comparison of general data of patients with liver cancer and those without liver cancer [(X̅ ±S ) or n].

Group	Cases (n)	Age (years)	Gender (Male/ Female)	BMI (kg/m^2^)
Patients with liver cancer	53	48.27±8.27	29/24	20.15±3.21
Patients without liver cancer	37	47.12±8.52	24/13	19.62±3.02
t/χ2 value	--	0.641	0.927	0.789
P value	--	0.523	0.336	0.432

Fifty-three patients were diagnosed as positive for primary liver cancer from serum MIF, of which eight had false positive results. Fifty-six patients were diagnosed as positive for primary liver cancer from serum GP73, of which Ten had false positive results. Forty-eight patients were diagnosed as positive for primary liver cancer from serum PIVKA-II, of which seven had false positive results. Fifty-two patients were diagnosed as positive for primary liver cancer from serum AFP-L3, of which nine had false positive results. Fifty-seven patients were diagnosed as positive for primary liver cancer from serum AFP, of which seven had false positive results. Fifty-three patients were diagnosed as positive for primary liver cancer from the combined detection of five serological tumor markers, of which one had a false positive result. Table-II.

**Table-II:** Diagnosis results of 5 serological tumor markers for primary liver cancer (single and combined detection).

Detection	Positive	False positive
MIF	53	8
GP73	56	10
PIVKA-II	48	7
AFP-L3	52	9
AFP	57	7
Combined detection	53	1

Serum MIF for the diagnosis of primary liver cancer had a sensitivity of 84.91%, a specificity of 78.38% and an accuracy of 82.22%. Serum GP73 for the diagnosis of primary liver cancer had a sensitivity of 86.79%, a specificity of 72.97% and an accuracy of 81.11%. Serum PIVKA-II for the diagnosis of primary liver cancer had a sensitivity of 77.36%, a specificity of 81.08% and an accuracy of 78.89%. Serum AFP-L3 for the diagnosis of primary liver cancer had a sensitivity of 81.13%, a specificity of 75.68% and an accuracy of 78.89%. Serum AFP for the diagnosis of primary liver cancer had a sensitivity of 83.02%, a specificity of 81.08%, and an accuracy of 82.22%. The combined diagnosis of 5 serological tumor markers for primary liver cancer had a sensitivity of 98.11%, a specificity of 97.30%, and an accuracy of 97.78%. And combined diagnosis had higher sensitivity, specificity and accuracy than the single diagnosis, with the difference statistically significant (P<0.05). Table-III.

**Table-III:** Comparison of the diagnostic value of 5 serological tumor markers for primary liver cancer (single and combined detection).

Detection	Sensitivity (%)	Specificity (%)	Accuracy (%)
MIF	84.91(45/53)	78.38(29/37)	82.22(74/90)
GP73	86.79(46/53)	72.97(27/37)	81.11(73/90)
PIVKA-II	77.36(41/53)	81.08(30/37)	78.89(71/90)
AFP-L3	81.13(43/53)	75.68(28/37)	78.89(71/90)
AFP	83.02(44/53)	81.08(30/37)	82.22(74/90)
Combined detection	98.11(52/53)^abcde^	97.30(36/37)^abcde^	97.78(88/90)^abcde^

**Note:** Compared with serum MIF, ^a^P< 0.05; compared with serum GP73, ^b^P< 0.05; compared with serum PIVKA-II, ^c^P< 0.05; compared with serum AFP-L3, ^d^P< 0.05; compared with serum AFP, ^e^P<0.05.

## DISCUSSION

Primary liver cancer is one of the common malignant tumors in recent years, with the incidence increasing year by year and no typical clinical symptoms in the early stage. Most patients are in the advanced stage at the time of diagnosis, and most need surgical treatment, but there are poor prognosis, high recurrence rate and metastasis rate, which seriously reduce the quality of life and survival time and threaten human health.^6^,^7^ Therefore, early diagnosis of primary liver cancer is of great significance to the prognosis and survival of patients. Detection of serum markers is an important means of early diagnosis of primary liver cancer. However, single biomarker detection has certain limitations, and it cannot detect all patients with liver cancer. Combined detection of multiple tumor markers can effectively improve the diagnostic efficiency and make up for the deficiency of single detection.^8^

AFP is an important serological indicator of liver cancer and a glycoprotein, with the synthesis and degradation of sugar chains completed inside the cell. The structure of the glucose chain is heterogeneous, which can be used as a specific index for clinical diagnosis of liver cancer, and has good diagnostic value for primary liver cancer. However, it can also be elevated in some patients with chronic hepatitis B and cirrhosis, lacking certain specificity as a diagnostic indicator of liver cancer.^9^ AFP-L3 is produced in liver cancer cells and is AFP heteroplastic. As a malignant tumor biomarker, it has high specificity for hepatocellular carcinoma, so it can become the most valuable marker for the diagnosis of liver cancer, contributing to the differentiation of liver disease and the diagnosis of liver cancer.^10^ GP73 is a transmembrane protein that exists in the Golgi apparatus and is expressed in epithelial cells of various human tissues. In patients with liver disease, its expression is significantly higher than that of normal people, which can be an effective serum marker for the diagnosis of primary liver cancer and help to improve the detection rate of patients with primary liver cancer.^11^ MIF is produced by activated T lymphocytes and plays a very important role in promoting the occurrence and development of tumors. It can limit the excessive phagocytosis of macrophages and can inactivate the human tumor suppressor gene p53. It also has the ability to promote tumor progression, which can promote the continuous regeneration of tumor blood vessels and induce tumor formation.^4^,^12^ PIVKA-II is synthesized by the liver and is abnormal prothrombin, without clotting activity. It is related to the abnormal metabolism of vitamin K in liver cancer cells and the lack of prothrombin precursors.^13^,^14^

At present, among relevant reports concerning primary liver cancer, most are the single detection of GP73, PIVKA-II, AFP-L3, AFP or other factors or the combined detection of 2 or 3 factors, and the combination of AFP and other serological indicators, including AFP+AFP-L3,^15^ AFP+PIVKA-II, AFP+GP73, AFP+AFP-L3+PIVKA-II.^16^,^17^ There are few related pieces of literature about MIF and primary liver cancer, and the direction of combined diagnosis of primary liver cancer by 5 related factors of MIF, GP73, PIVKA-II, AFP-L3, and AFP has not been clear yet. From An XG et al.^12^, serum MIF in patients with primary liver cancer was significantly increased, and gradually increased with the progress of the tumor, which might promote the growth and deterioration of liver cancer cells. From Zhang JN et al.^18^ PIVKA-II could be used as an important indicator for the early screening of liver cancer, and the combined detection with AFP could improve the sensitivity and specificity of the diagnosis of primary liver cancer. From Lv CY et al.^19^ serum AFP, AFP-L3 and PIVKA-II had very high clinical diagnostic value for primary liver cancer, and the combined detection of the 3 indicators could significantly improve the diagnostic efficiency for primary liver cancer. He J et al.^20^ Pointed out that AFP had high sensitivity in diagnosing primary liver cancer, and AFP-L3 had high specificity in diagnosing primary liver cancer. A single detection of AFP or AFP-L3 had a very high differential diagnosis value for liver cancer and non-liver cancer, and the combined detection of these two could help reduce the clinical misdiagnosis rate. From the results of this study, for the diagnosis of primary liver cancer, the single detection of serum MIF, GP73, PIVKA-II, AFP-L3 or AFP had a sensitivity of 84.91%, 86.79%, 77.36%, 81.13% and 83.02%, respectively; a specificity of 78.38%, 72.97%, 81.08%, 75.68% and 81.08%, respectively; an accuracy of 82.22%, 81.11%, 78.89%, 78.89% and 82.22%, respectively, indicating that all the five serological tumor markers had certain diagnostic value and could be used for the early diagnosis of primary liver cancer. However, the sensitivity, specificity and accuracy still needed to be improved, indicating that single detection for the diagnosis of primary liver cancer still had certain limitations. In this study, in the diagnosis of primary liver cancer, the combined detection of five serological tumor markers had a sensitivity of 98.11%, a specificity of 97.30% and an accuracy of 97.78%, and the sensitivity, specificity and accuracy of combined diagnosis were significantly higher than those of the single diagnosis, indicating that the combined detection of multiple serological indicators could effectively improve the sensitivity, specificity and accuracy of the diagnosis compared with the single detection of the serological index. It is suggested that the combined detection of five serological tumor markers had a higher diagnostic value for primary liver cancer, which will provide a reliable reference for clinical diagnosis and treatment, and can be used for clinical reference and application.

### Limitations of the study

It includes small sample size. in this stud. Large sample size is required to confirm the diagnostic value of 5 serological tumor markers such as MIF and GP73 for primary liver cancer, so as to provide more effective guidance for clinical diagnosis.

## CONCLUSION

Serum MIF, GP73, PIVKA-II, AFP-L3 and AFP all have certain diagnostic value for primary liver cancer, and the combination of five serological tumor markers for detection can significantly improve the sensitivity, specificity and accuracy of the diagnosis of primary liver cancer, with a higher diagnostic value.
